# *Agrobacterium tumefaciens*: A Bacterium Primed for Synthetic Biology

**DOI:** 10.34133/2020/8189219

**Published:** 2020-05-26

**Authors:** Mitchell G. Thompson, William M. Moore, Niklas F. C. Hummel, Allison N. Pearson, Collin R. Barnum, Henrik V. Scheller, Patrick M. Shih

**Affiliations:** ^1^Joint BioEnergy Institute, Emeryville, CA, USA; ^2^Environmental Genomics and Systems Biology Division, Lawrence Berkeley National Laboratory, Berkeley, CA, USA; ^3^Department of Plant Biology, University of California-Davis, Davis, CA, USA; ^4^Department of Plant and Microbial Biology, University of California-Berkeley, Berkeley, CA, USA; ^5^Genome Center, University of California-Davis, Davis, CA, USA

## Abstract

*Agrobacterium tumefaciens* is an important tool in plant biotechnology due to its natural ability to transfer DNA into the genomes of host plants. Genetic manipulations of *A. tumefaciens* have yielded considerable advances in increasing transformational efficiency in a number of plant species and cultivars. Moreover, there is overwhelming evidence that modulating the expression of various mediators of *A. tumefaciens* virulence can lead to more successful plant transformation; thus, the application of synthetic biology to enable targeted engineering of the bacterium may enable new opportunities for advancing plant biotechnology. In this review, we highlight engineering targets in both *A. tumefaciens* and plant hosts that could be exploited more effectively through precision genetic control to generate high-quality transformation events in a wider range of host plants. We then further discuss the current state of *A. tumefaciens* and plant engineering with regard to plant transformation and describe how future work may incorporate a rigorous synthetic biology approach to tailor strains of *A. tumefaciens* used in plant transformation.

## 1. Introduction

*Agrobacterium tumefaciens* is a soil-dwelling plant pathogen that has been well studied due to its unique natural ability to transform many species of plants and other eukaryotes. Naturally, *A. tumefaciens* pursues a pathogenic lifestyle by transforming plants with an ssDNA that induces the plant to form a tumor (termed a gall) via the synthesis of phytohormones. Additionally, the bacterium coerces the plant to synthesize compounds known as opines from plant metabolites whose use as a nutrient source is restricted to the pathogen [1]. The DNA that is transferred to the host (termed T-DNA) is encoded on a large virulence plasmid (pTi), which also encodes most of the genes necessary to carry out pathogenic functions [2]. These virulence (or *vir*) genes encode the machinery required to process T-DNA and translocate it efficiently into the plant cell nucleus, where its tumorigenic genes can be integrated into the chromosome or transiently expressed [3].

Very soon after the molecular details of *A. tumefaciens* pathogenesis were elucidated, its value as a biotechnological tool was realized. Researchers were able to introduce transgenes into plants by replacing the oncogenic loci with other genetic elements [4]. Since that initial leap, *A. tumefaciens* has become the single most important tool in agricultural biotechnology. A great amount of optimization has been required to increase the efficiency by which *A. tumefaciens* introduces DNA into the host and expand the host range [5]. Many external factors dramatically influence *Agrobacterium*-mediated transformation (AMT), including the age of the plant, tissue type, length of agroinfiltration, day-night cycling, and method of agroinfiltration (Table 1). However, modulating the pathogenicity of the bacterium itself has been proven extremely useful in broadening the utility of *A. tumefaciens* as a tool for both transient expression and stable genetic integration.

**Table 1:** Examples of parameters optimized to improve AMT outcomes.

Variable	Key points of optimization from study	Citation
Day-night cycles	Best Arabidopsis transient expression was shown in plants grown in short day conditions, kept in 85-90% relative humidity for 24 h after infiltration, using Agrobacterium strain LAB4404, and addition of 0.01% Triton X-100 to infiltration media	[140]
Plant tissue pretreatment	Highest transformation efficiency of Sorghum bicolor L. by heat treating immature embryos at 43°C for 3 min then cooling to 25°C prior to infection	[141]
Cultivar optimization	Found optimal infiltration of Glycine max (soy) to occur in cultivars Jack Purple or Tianlong 1 with Agrobacterium suspension of $\text{O}{\text{D}}_{650}=0.6$ containing 154.2 mg/L dithiothreitol and cocultured for 5 days	[142]
Plant age	Found optimal infiltrations of Brassica oleracea var. botrytis (cauliflower) to occur in 7-day-old seedlings, 50 *μ*M acetosyringone, 3-day cocultivation at 22°C, using agro strain GV2260	[143]
Antioxidant addition	Found the addition of antioxidants (lipoic acid or glycine betaine) increased transformation efficiency in Citrus aurantifolia Swingle (Mexican lime)	[144]
Duration of agroinfiltration	Performed transient expression in the adaxial epidermis and mesophyll of onion scales. Found the agroinfiltration durations and OD600 to be the largest factors (best: 43.87% transformation efficiency with conditions of 72 h and OD_600_ of 0.10). Also found all components of their infiltration media to be necessary (1 component missing reduced efficiency by 30-41%)	[145]
Method of agroinfiltration	Found use of 6 min sonication and 6 min vacuum infiltration (750 mmHg) provided highest efficiency (39.4%) in banana cv. Rasthali. This seems to be a common method for increasing transformation efficiency in rarely transformed plants (e.g., de Oliveira et al., 2008; Bakshi et al., 2011; Liu et al., 2005)	[146]

Nearly twenty years ago, Gelvin reviewed the biological basis of *A. tumefaciens* pathogenesis through the lens of genetic engineering [6]. They highlighted virulence factors and host proteins that could be modulated or engineered to affect the rates of AMT or broaden the host range of *A. tumefaciens*. Since that time, cheap, accurate DNA sequencing and tools to precisely edit genomes, such as CRISPR/Cas9, have spurred major shifts in the technology used to study biological systems. The field of synthetic biology, which seeks to rationally design and engineer biological systems to generate predictable, useful outcomes, has also rapidly matured. While perhaps most prominently utilized in the field of metabolic engineering, synthetic biology has also been invoked to rewire other complex biological phenomena such as nitrogen fixation [7], respiration through changes in membrane viscosity [8], and altering the plant cell wall [9]. This engineering is predicated on the existence of well-characterized DNA elements that can precisely control the expression of genetic circuits; however, we still lack the DNA parts needed to enable complex and fine-tuned engineering efforts in *Agrobacterium*.

In this review, we focus on recent advances in the understanding of *Agrobacterium*-host interactions that may serve as targets for synthetic biology approaches to increase the potency of *A. tumefaciens* as a tool for precisely engineering a broad range of plants. Furthermore, we highlight technological advances in the delivery of T-DNA to host cells, as well as the current repertoire of genetic tools available to control gene expression in *A. tumefaciens*. Finally, we discuss which areas the field may focus on to improve the control of *Agrobacterium*-based genetic circuits in the future.

## 2. Engineerable Targets of *A. tumefaciens*

*Agrobacterium* pathogenesis of plant tissues progresses in a stepwise manner, requiring attachment to the plant cell surface, small molecule perception for virulence induction, T-DNA transfer into the host cell, nuclear import of the proposed T-complex (the assemblage of a T-DNA covalently attached to a VirD2 molecule and putative associations with other *vir* proteins), and stable integration into the host genome. Each step represents a potential bottleneck. Here, we will briefly cover the basic biological steps of *A. tumefaciens* pathogenesis that may lend themselves to useful engineering.

### 2.1. Induction of *vir* Genes

In natural settings, *A. tumefaciens* pathogenesis begins when the bacterium migrates towards the site of wounded plants, attracted to a myriad of phenolics and sugars [10, 11]. This chemotaxis machinery has been reviewed in depth elsewhere [12, 13], and although flagella mutants do show reduced virulence [14], motility towards the plant is generally thought to be irrelevant during laboratory transformation protocols, where plant material is directly infiltrated with bacterial culture. However, many of the signals that are proposed to draw *A. tumefaciens* to the plant also induce the *vir* genes required to initiate pathogenic DNA transfer into the host plant.

The *vir* genes are regulated by the master regulator two-component system (TCS) VirA (the sensor kinase) and VirG (the response regulator), which is able to integrate multiple environmental signals. The VirA sensor is thought to be directly activated by plant-derived phenolic compounds, such as acetosyringone (AS) [15] but can indirectly sense plant-derived monosaccharides via the aid of the chromosomally encoded periplasmic sugar-binding protein ChvE [16] and pH through the chromosomally encoded ChvG/I TCS [17]. These environmental factors contribute to the induction of the *vir* genes located on the pTi plasmid and also to an increase in the copy number of pTi itself, ensuring efficient synthesis of T-DNA. The molecular details of these pathways have been extensively reviewed elsewhere [12, 13, 18].

Much research has focused on optimizing *vir* gene induction by VirG, often with the addition of exogenous AS or other media components that are sensed directly or indirectly by VirA. A recent work in rice showed that for large (50 kb) T-DNA insertions, cultures of *A. tumefaciens* could require as much as 24 hours of AS induction before robust T-DNA formation could be observed [19]. In another recent study, transient expression of T-DNA was optimized in *Arabidopsis* seedlings in a method termed AGROBEST [20]. A key finding of the study was that buffered infection media with pH of 5.5 increased transient *β*-glucuronidase (GUS) activity 20-fold. A further work in Brazilian wheat and the algae *Dunaliella* has optimized levels of glucose, AS, and pH for more efficient transformation [21, 22]. These studies are just a few examples of how titrating the signal inputs that activate VirG can shape the outcome of transformation. Beyond the VirA/G TCS, a more recent work has also implicated the role of small noncoding RNAs (ncRNAs) in the regulation of *A. tumefaciens* virulence and has been recently reviewed [23]. One example of the role of ncRNAs play in *A. tumefaciens* virulence is a work demonstrating that the chromosomally encoded ncRNA *AbcR1* negatively regulates ChvE, amongst other nutrient transporters that may affect *vir* gene induction [24, 25]. As work on these RNAs matures, more insight will be gained into how these systems may be engineered to optimize AMT.

Almost universally, AMT relies on induction of the *vir* genes by the native regulatory machinery. This reliance on natural regulation leaves the system susceptible to host interference with VirG activation and may disallow for specific titration of key *vir* genes. It has been demonstrated that when the constitutively active VirG mutant *virG*N54D was overexpressed on a compatible plasmid, transformation efficiency increases in a variety of plants and even broadening the host range to include the legume *Melilotus alba* and the flowering plant *Heteromorpha arborescens* [26, 27]. This work suggests that high-level constitutive activation of the *vir* genes is a formidable strategy for *Agrobacterium*-mediated gene transfer. Completely decoupling native regulation from *vir* gene expression could reduce the likelihood of plant repression of gene expression and allow researchers to precisely tune *vir* gene levels to specific experimental needs [28, 29].

### 2.2. Attachment to Plant Surfaces

The attachment of *A. tumefaciens* to both abiotic and biotic surfaces has also been reviewed extensively elsewhere [30, 31]. In a natural environment, it is commonly believed that *A. tumefaciens* must first establish a physical interaction with a host plant in order to then initiate the transfer of T-DNA. Attachment to surfaces is thought to occur in a stepwise fashion: first a potentially transient reversible attachment is established, followed by irreversible attachment to a surface. Flagellar motility facilitates initial surface contact, where reversible attachment is potentially mediated by a variety of pili (Ctp and conjugative) or protein adhesins [30]. Subsequent irreversible attachment is mediated by exported polymers, primarily cellulose and unipolar polysaccharide (UPP) [31]. However, in common laboratory models, these attachment structures are largely unnecessary for virulence [30], likely due to the large amount of bacteria that are directly added to already wounded tissues in laboratory conditions. It has also been noted that none of these attachment structures are coded for on the virulence plasmid and the *att* genes that are on pAtC58 which are not essential for virulence [30]. Despite these observations, it is still unknown whether attachment to the plant surfaces may play an important role in expanding the host range of *A. tumefaciens*. A recent work demonstrated that addition of exogenous cellulose to barley callus cultures not only increased bacterial adherence to callus cells but also increased GUS staining by 50% compared to conventional conditions [32]. Given such observations, it is valuable to have control over known attachment structures and continue to search for novel mediators of *Agrobacterium*-plant interactions.

In *A. tumefaciens*, the biosynthesis of both cellulose and UPP are posttranslationally regulated by the global secondary messenger cyclic diguanylate (c-di-GMP), with higher intracellular levels of c-di-GMP generally resulting in greater polysaccharide production [33, 34]. The levels of c-di-GMP are most often controlled by two competing classes of enzymatic activities: c-di-GMP forming diguanylate cyclases (DGCs) and c-di-GMP hydrolyzing phosphodiesterases (PDEs) [35]. *A. tumefaciens* has numerous enzymes that have DGC or PDE activity, in some cases in the same protein [36]. A recent work has shown that the activity of the bifunctional DGC/PDE enzyme DcpA was also curiously dependent on the pteridine reductase PruA [36]. The authors showed that when bound to a novel pteridine-derived cofactor, the pterin-binding family protein PruR enhances the PDE activity of DcpA. Disruption of pterin biosynthesis resulted in *A. tumefaciens* that formed large clumps in liquid culture, likely due to an increased synthesis of UPP. This work suggests that engineers could control the “stickiness” of *A. tumefaciens* by titrating the relative levels of pterin biosynthesis as well as expression of DcpA.

An active area of research that remains mostly unexplored is the interaction between *A. tumefaciens* and the plant cell wall. To date, there are no proven direct interactions between specific cell wall moieties and specific attachment appendages in *A. tumefaciens*. However, it has recently been shown that multiple glycoside hydrolases play a role in *A. tumefaciens* virulence of tomato and *Bryophyllum daigremontianum* [37]. By screening deletion mutants of all 48 predicted glycoside hydrolases of *A. tumefaciens*, Mathews and colleagues showed that mutants in enzymes predicted to degrade polygalacturonic acid, xylan, and arabinogalactans were all less virulent than wild-type *A. tumefaciens*. Furthermore, mutants in multiple enzymes predicted to possess *β*-glycosidase activity and an arabinofuranosidase that may cleave arabinose from the ends of polysaccharide strands were also shown to be less virulent. As mutation to any of the identified hydrolases nearly completely abolished tumor formation in both host plants, these results suggest that cell wall degradation or polysaccharide catabolism may play key steps in establishing pathogenicity. The logical next step may be to explore additional cell wall degradative enzymes to test whether they can further increase the efficiency of T-DNA transfer.

### 2.3. T-DNA Generation and Transfer

Upon induction by the VirA/G TCS, *A. tumefaciens* begins the process of creating ssDNA T-DNA strands destined for export to host cells via a type IV secretion system (T4SS) encoded by the *virB* cluster of genes and *virD4*. The *A. tumefaciens* T4SS has served as a model for other bacterial T4SSs, and both its structure and function have been reviewed extensively [38]. However, the T4SS itself has rarely been the target of efforts to increase the efficiency of T-DNA transfer. Other steps in T-DNA generation have been extensively modified to augment transformation efficiency. T-DNA strand formation largely relies on the formation of a DNA nicking complex consisting of the proteins VirD1, VirD2, VirC1, and VirC2 [39]. VirD2 initially acts as an endonuclease that nicks the *cis*-acting left and right borders (termed LB and RB, respectively) to generate the T-DNA [3]. Subsequently, VirD2 is covalently bonded to the 5${}^{\prime }$-end to form a nascent T-strand, which is critical for stability, export, and future localization [3]. VirC1 binds to a *cis*-acting site proximal to the T-DNA border termed *overdrive* [40] and in association with VirC2 increases the production of T-strands [39]. An exceptional work done by Atmakuri et al. demonstrated that VirC1 initiates the assembly of the relaxosome and dramatically stimulates the production of T-DNA strands in conjunction with VirC2 [39]. T-DNA strand generation is also influenced by the copy number of the virulence plasmid, which is increased by VirA/VirG when stimulated by plant wound signals [41]. In later sections, we will discuss in detail how binary vectors have been designed to optimally produce T-DNA strands.

Beyond the canonical *vir* genes, it has recently come to light that the membrane composition of *A. tumefaciens* can play an important role in shaping the pathogenicity of the bacterium. When potato is infected with *A. tumefaciens* that lack ornithine lipids, tumors form much more rapidly [42]. To date, the specific physiological function of ornithine lipids in bacteria has not been precisely defined, though it is believed to be associated with resistance to abiotic stresses. It has yet to be understood whether the lack of ornithine lipids may increase *A. tumefaciens* resistance to plant immunity or signaling, or rather increase the ability for *A. tumefaciens* to transfer T-DNA [42]. Another work has shown that the levels of lysyl-phosphatidylglycerol (L-PG) in the membrane can dramatically alter *A. tumefaciens* virulence [43]. Researchers showed that mutation of the L-PG hydrolase AcvB increased the levels of L-PG in the membranes ~10-fold, which dramatically reduced T-DNA transfer in a transient *A. thaliana* GUS-assay. The precise mechanism by which the level of L-PG affects virulence is still unknown. It is possible that changes in membrane composition reduce the ability of the VirA/G TCS to activate *vir* gene expression, though it is just possible that these changes make it difficult for the T-pilus to properly assemble. As more light is shed on the role of how membrane composition affects virulence, rational engineering of lipid compositions could become another means of controlling *Agrobacterium*-plant interactions.

### 2.4. T-DNA Trafficking in the Host

The assembly of the T-complex and subsequent trafficking to the nucleus has been reviewed extensively elsewhere [44, 45]. It has been suggested that once the T-DNA-VirD2 complex enters the host cell, it is rapidly coated by VirE2, which serves partly to protect the ssDNA from nuclease-mediated degradation [45]. Both VirD2 and VirE2 contain predicted nuclear localization sequences (NLSs) and interact with host factors that guide the T-complex to the chromatin [45]. VirD2 is known to interact directly with the nuclear localization machinery component importin *α* [45]. Initially, it was believed that VirE2 requires additional factors from the plant host, termed VirE2 Interacting Proteins (VIPs), to act as intermediaries with importins [45]. However, this view has been challenged by a work that used yeast two-hybrid (Y2H) assays to show that VirE2 can interact with multiple importin *α* proteins of *A. thaliana*. This same research also showed that *A. thaliana* mutants in IMP*α*-4 are resistant to infection by *A. tumefaciens*, suggesting that IMP*α*-4 may facilitate VirE2 nuclear translocation [46]. Further research demonstrated that VIP1, which had been believed to assist VirE2 nuclear translocation, was dispensable for *A. thaliana* transformation by multiple *A. tumefaciens* strains [47]. The putative roles VIPs may play in *A. tumefaciens* virulence are discussed in detail in later sections. The ambiguity of how the T-DNA complex is imported into the nucleus extends to the host-specificity factor VirE3, which was thought to be able to mimic VIP1 function by facilitating importin interaction, thus allowing for infection in plants with low or absent VIP1 expression [48]. However, a recent work has shown that VirE3 localizes to the plasma membrane of *N. benthamiana* where it colocalizes with VirE2 and is required to protect the T-DNA, calling in to question its function as a VIP1 mimic [49]. Given that the current data conflict on the mechanisms of T-DNA trafficking, more work likely must be done before these processes can be rationally engineered to optimize AMT.

Once the T-complex has entered the nucleus, it has been hypothesized that the VirE2 coating is degraded for efficient transient expression or chromosomal integration to occur. VirF is believed to be another host-specificity factor that plays an important role in the degradation of the VirE2 coating [50]. VirF acts as an F-box protein within the nucleus and aids in the ubiquitination and degradation of both VIP1 and VirE2, though its specific role in pathogenesis may be debatable given our current understanding of T-complex nuclear translocation [50]. VirF itself is also proteolytically degraded, which can be mitigated by binding to an additional virulence effector, VirD5 [51]. VirF is required for infection in some plants, but not others—presumably due to other host factors filling the role of VirF [52]. Furthermore, in maize, its expression can actually inhibit the transfer of T-DNA, highlighting its variable role in AMT [52]. VirD5 has also been shown to be toxic to host cells, as it can cause chromosome missegregation in yeast by stimulating the activity of Aurora family kinase Ipl1, which leads to detachment between the kinetochore and mitotic spindles [53]. The variable effects of both VirF and VirD5 both highlight the importance of the ability to precisely regulate individual *vir* gene expression in future *A. tumefaciens* engineering efforts.

### 2.5. Host Evasion and Resistance

Like many plant pathogens, *A. tumefaciens* has evolved multiple mechanisms to evade and subvert the immune responses of plant hosts. This manipulation of the host response has also been reviewed extensively [3, 45, 54]. Wounded plants often produce toxic phenolic compounds, which in addition to stimulating the expression of the *vir* genes can also be toxic to *A. tumefaciens* [55]. An early work demonstrated that some pTi plasmids harbor VirH homologs that code for cytochrome p450 monooxygenases, which can be induced in the presence of phenolics and are capable of catabolizing toxic concentrations of ferulate [55]. In response to many bacterial pathogens, plants undergo a hypersensitive response (HR) that causes tissue necrosis to halt the spread of infection, producing reactive oxygen species such as H_2_O_2_ in the process [56]. Though *A. tumefaciens* does not induce HR in its native hosts, the ability of catalase mutants to form tumors in the nonnative host *Kalanchoe* was compromised [56]. These results indicate that engineering strains of *A. tumefaciens* that can perceive and respond to plant defenses may be critical as researchers try to expand its host range.

Beyond dealing with physical and chemical defenses imposed by the host, *A. tumefaciens* has the ability to actively modulate the host response by reprogramming host transcription. Host specificity factor VirE3 was shown to bind to the *A. thaliana* transcription factor (TF) pBrp and assist in its translocation to the nucleus, where it stimulates the expression of the F-box protein VBF [57]. Previously, VBF had been shown to perform the same function as VirF, enhancing the ability of *A. tumefaciens* to transform plants [50]. VirF has also been shown to modulate host transcription in *A. thaliana* by targeting the TF VFP4 for degradation via the proteasome [58]. Mutants in VFP4 showed reduced expression of multiple genes associated with bacterial defense, suggesting *A. tumefaciens* may actively repress the plant defenses. As our understanding of how *A. tumefaciens* modulates the plant-host response improves, it is foreseeable that specific proteins could be engineered into the bacterium to suppress plant bacterial defenses. These proteins need not necessarily be derived from any *Agrobacterium* species, but rather could draw upon the numerous bacterial effectors that are known to inhibit plant immunity [59].

## 3. Engineering Targets of Host Plants

During its pathogenesis, *A. tumefaciens* encounters a variety of plant host factors that can limit or aid plant transformation efficiency. Plant regeneration post T-DNA integration is yet another significant hurdle for many agronomically important plant species and genotypes. Here, we discuss known host factors that modulate AMT, as well as recent engineering strategies that increase plant regeneration efficiency.

### 3.1. Virulence Induction and Attachment

As discussed previously, small molecules derived from wounded host tissue act as potent inducers of *A. tumefaciens* virulence. Beyond the canonical inducer molecules, low environmental calcium content has been shown to influence bacterial polar attachment to root hairs [30]. Low-calcium content in *A. tumefaciens* suspension culture upregulates UPP production and polar attachment [30], while calli propagated on low calcium media also increased transformation efficiency [60]. Importantly, calcium also modulates plant cell wall architecture by crosslinking homogalacturonan pectic polysaccharides in the so-called “egg box conformation” and increases the rigidity of the cell wall [61]. As the cell wall is a barrier to pathogen attack and evidence from both *A. tumefaciens* and the plant host indicates that cell wall loosening is important for transformation, much effort has gone into identifying the genetic basis for plant resistance to *A. tumefaciens*.

Forward genetic screening for *A. thaliana* mutants resistant to *Agrobacterium* transformation (*rat*) identified a number of cell wall synthesis genes including a mannan synthase (*rat4*), *β*-expansin (*ratT18*), and arabinogalactan protein (AGP) or extensin-type glycoproteins (*rat1* and *rat3*) [6, 62]. These enzymes may be important for cell wall loosening and also may release sugars that synergistically induce *vir* genes together with plant-derived phenolic compounds. Interestingly, the lysine-rich arabinogalactan protein 17 (AGP17/*rat1*) is required for polar attachment of *A. tumefaciens* to plant tissue and AMT [63]. The related alpha-Proteobacterium *Rhizobium leguminosarum* is able to bind gum arabic (a pure AGP fraction from *Acacia senegal*) and purified AGP from pea roots *in vitro* and form polarized arrays but is unable to form polar attachments with hemicelluloses or pectins [64]. Furthermore, chemical disruption of AGPs prevented the polar attachment *R. leguminosarum in vitro* [64]. Many AGPs including AGP17/*rat1* contain glycosylphosphatidylinositol (GPI) lipid anchors that tether the glycoprotein to the outer leaflet of the plasma membrane. Conceivably, binding of periplasmic GPI-anchored proteins brings the bacterium into close proximity with the plant plasma membrane for pili formation and conjugation. Some evidence has shown that AGPs may also participate in endocytosis-driven mechanisms in pollen tube self-incompatibility [65] and microelement accumulation [66], which could potentially be relevant for endocytic trafficking of *A. tumefaciens* effectors. Though both *A. tumefaciens* and *R. leguminosarum* form polar attachment structures in response to AGP cell surface epitopes, the underlying mechanisms and biology are not well understood. As our knowledge of plant mediators of bacterial attachment expands, new engineering strategies may emerge. Beyond the natural attachment appendages encoded within *A. tumefaciens*, it may be feasible to engineer systems from other bacteria with better characterized plant interactions.

### 3.2. T-DNA Transfer and Nuclear Import

Currently, very little is known about plant host proteins involved in assisting T-DNA and effector protein delivery into the plant cell. However, Y2H assays using VirB2 as bait identified a group of reticulin-like B proteins (RTNLB) and a RAB GTPase as reciprocally interacting proteins associated with the T-pilus type IV secretion system [67]. *A. thaliana rtnlb3* loss of function mutants had decreased levels of transient T-DNA expression [68], while lines overexpressing either RTNLB3 or RTNLB8 were hypersusceptible to both *A. tumefaciens* and *Pseudomonas syringae* DC3000 [68]. A recent work has also shown that RTNLB4 may participate in the VirB2 and ELF18 peptide-induced defense against *A. tumefaciens* [69]. RTNLB proteins likely have other roles in plants but may be commonly exploited by pathogenic bacteria for pathogenesis.

As previously discussed, nuclear import of T-DNA is largely mediated by VirE2 and VirD2, which contain nuclear localization signals, but have been shown to interact with a number of host factors. The most contested of theseis VIP1. In plants, VIP1 is a transcriptional activator of stress response with reported roles in mechanical wounding, salt stress, hypoosmotic stress, *Botrytis* infection, sulfur deficiency, and purportedly *agrobacterium* pathogenesis [70–75]. VIP1 normally resides in the cytoplasm and localizes to the nucleus in response to stress to regulate transcription [45, 70, 74, 76]. VIP1 interacts with VirE2 *in vitro* and forms complexes *in vivo* in the plant cytoplasm [47, 75, 77]. It was reported that MAPK3 phosphorylation of VIP1 at Ser79 may enhance T-DNA nuclear import via interactions with VirE2 [74]. However, *vip1* mutant plants are not impaired in AMT efficiency and subsequent rigorous studies have seriously challenged the VirE2 nuclear import capabilities of VIP1 and the Ser79 phosphorylation site [47]. More recently, VIP1 nuclear localization during mechanical stress was shown to be regulated by dephosphorylation at Ser35 and Ser115 by Protein Phosphatase 2A (PP2A) [73]. Alanine substitution at these sites triggered constitutive nuclear localization of VIP1 [73]. From this perspective, it would be interesting to test whether alanine-substituted VIP1 could increase AMT. However, further research into if VIP1 is necessary before it can be incorporated into rational engineering strategies applied to AMT.

Other factors influencing nuclear import include phosphorylation of VirD2. Dephosphorylation of VirD2 by PP2C has been shown to impair T-DNA nuclear import [78]. Therefore, engineering strategies that either reduce host PP2C expression [79] or include a phosphatase resistant VirD2 phospho-mimic may further increase nuclear import efficiency.

### 3.3. T-DNA Integration

After nuclear import, the T-complex is proposed to be disassembled by SCF complex E3 ubiquitin ligase-mediated proteolysis to expose the T-DNA strand for integration into the host genome. To date, many different host factors that affect T-DNA integration and transgene expression have been identified but can generally be grouped into three related categories including (1) DNA repair, (2) nonhomologous end joining, and (3) chromatin remodeling.

Many mutants that are sensitive to DNA damage or defective in nonhomologous end joining have increased transformation efficiency [80–83]. Presumably, attenuated double stranded DNA break repair increases the probability of T-DNA integration. Further evidence suggests that either limiting nonhomologous recombination (NHR) or promoting homologous recombination (HRec) increases T-DNA integration. Multiple works have shown that Ku70, Ku80, Lig6, and XRCC4 are mediators of NHR that inversely affect DNA integration [84, 85]. VirE2 interacts with XRCC4 in Y2H assays and may have a function in titrating XRCC4 levels to favor T-DNA integration [82, 84]. Chromatin remodeling protein RAD54 is known to be important for HRec in both yeast and mammalian cells [86]. Expression of RAD54 in plants increased HRec T-DNA integration into a specific locus by 27-fold, and it was determined that chromatin remodeling in plants was the most limiting factor for HRec integration [87].

Alterations in chromatin structure influence DNA accessibility and the frequency of T-DNA integration into the host genome. Arabidopsis histone HTA-1 is a core H2A histone protein identified in a forward genetic mutant screen for plants resistant to *Agrobacterium* transformation (*rat5*) [88, 89]. Mutations in *hta-1/rat5* inhibit AMT while HTA-1 overexpression increases transformation efficiency in both *Arabidopsis* and rice [89–91]. Similarly, plants deficient in chromatin assembly factor (CAF-1) have higher levels of T-DNA integration, presumably due to increased accessibility of unbound DNA to invading T-DNA [81]. Lastly, the transcriptional activator VIP2 is also important for stable T-DNA integration and has been shown to influence chromatin structure by regulating histone expression [92]. However, overexpression of VIP2 did not increase T-DNA integration like HTA-1 [89, 90, 92]. Therefore, a future work that actively programs specific routes of DNA repair or chromatin remodeling can dramatically increase the efficiency and specificity of T-DNA integration.

### 3.4. Plant Defense and Stress Response

Transcriptomic and functional studies have shown that *A. tumefaciens* pathogenesis induces plant defense and cellular stress responses with concomitant downregulation of genes associated with plant growth [93–95]. Localized plant defense responses are activated through the perception of pathogen-associated molecular patterns (PAMPs) like flagellin, chitin, and EF-Tu, by specific receptor-like kinases (RLK) at the cell surface. EF-Tu is the major *Agrobacterium* PAMP recognized by plants resulting in PAMP-Triggered Immunity (PTI). EF-Tu is recognized by the EF-Tu receptor (EFR) and activates MAPK signaling cascades resulting in a robust defense response through the production of reactive oxygen species [96]. *A. thaliana efr* mutants are hypersusceptible to *Agrobacterium* infection, and transient T-DNA expression is greatly increased [96]. However, it is unknown whether stable T-DNA integration is also affected in *efr* mutants. Lastly, while *A. tumefaciens* elicit a formidable localized PTI response, hypersensitive response characteristic of effector triggered immunity (ETI) has not been reported.

Post infection, the expression of *A. tumefaciens* T-DNAs elicits a potent RNA silencing response by the plant host resulting in the accumulation of small interfering RNAs (siRNA) [97]. Production of different *cis* and *trans* acting 21-24 nt siRNAs by Dicer-like RNA endonucleases (DCLs) activates the RNA interference silencing complex (RISC) to mediate target mRNA degradation and DNA methylation [98]. T-DNA has been shown to be rapidly methylated during transient expression in infiltrated tobacco leaves [99]. Furthermore, transformation efficiency and T-DNA integration of 17 soybean genotypes were correlated with transgene methylation [100]. Correspondingly, chemical methylation inhibitors increased both T-DNA integration and transgene expression [100]. It is therefore not surprising that *A. thaliana* deficient in RNA interference are hypersusceptible to *A. tumefaciens* with increased transformation efficiency [97]. Successful natural pathogenesis of *A. tumefaciens* relies on a state of silencing suppression for the expression of oncogenes necessary for the progression of the disease. Similarly, the utility of *A. tumefaciens* as a plant engineering tool also relies on the efficient and stable expression of integrated genetic constructs. In practice, coexpression of viral silencing suppressor proteins such as P19 and P38 has commonly been used to quell siRNA response [101, 102]. Construct design incorporating 5${}^{\prime }$ and 3${}^{\prime }$ UTR sequences and introns has also been shown to reduce DNA methylation and transgene silencing [99].

Lastly, plant cellular stress also plays a role in the governing susceptibility to *A. tumefaciens. Arabidopsis* loss of function mutants in the mitochondrial porin protein Voltage-Dependent Ion Channel 1 (VDAC1) is resistant to *A. tumefaciens* infection at early steps prior to T-DNA transfer [103]. Plants overexpressing VDAC1 were hypersusceptible to infection with increased transient T-DNA expression but not stable integration [103]. The authors speculate that VDAC1 may regulate cellular and metabolic stress homeostasis to prevent organelle ROS production and associated apoptosis. Clearly circumventing plant defense is critical for efficient AMT. Future engineering efforts in this area will increase AMT efficiency, host range, and transgene stability.

## 4. AMT Engineering to Date

Since the initial creation of the binary vector system, tremendous progress has been made to generate plasmids that are both easy to manipulate, and yield highly efficient AMT in a variety of plants. Though binary and ternary vector development has somewhat dominated AMT engineering, there have been preliminary efforts to characterize genetic parts, as well as rationally engineer the *A. tumefaciens* genome to be a better chassis for T-DNA transfer. Beyond *A. tumefaciens*, there has recently been extraordinary work done to dramatically increase the efficiency of plant regeneration. Together these advances hint at a promising future for AMT in more plants and with greater precision.

### 4.1. Binary and Ternary Vector Engineering

Synthetic biology in *A. tumefaciens* began with the outsourcing of the T-DNA portion of the Ti-plasmid into a smaller vector, while maintaining the *vir* genes on a separate helper plasmid. This “binary vector” design made it possible to easily modify the genes transferred as part of the T-DNA region, and AMT has since become a standard tool in the transformation and molecular study of a wide variety of plant and fungal species. A multitude of binary vector systems have been developed and extensively reviewed [104–106]; however, the methodology is still not flawless. Problems with AMT include low transformation frequency, genome integration of multiple T-DNA copies, and genome integration of binary vector backbone, with the latter two often coinciding. [107]. Multiple T-DNA integrations can lead to silencing of the gene of interest [108], and transgenic plant lines harboring bacterial selection markers from backbone integration are a matter of public concern because horizontal gene transfer from transgenic plants to bacteria has been shown in recombination-favoring laboratory settings [109].

To address these issues, efforts have focused on minimizing binary vector size and utilizing standardized cloning techniques to speed up binary vector design, such as Golden Gate cloning, Gibson assembly, and the Gateway technology [110, 111]. The high copy binary vectors pBBR1 and pVS1 show increased transformation frequency after screening in different *A. tumefaciens* strains [100, 112]. Still, while plasmids with low copy Origin of Replication (ORI), like pRi, have a lower vector copy number and reduced transformation frequency, the frequency of single-copy integrations is significantly higher than the previously mentioned ORIs (Table 2) [113]. Anand et al. combined the strategies of size minimization and ORI change and induced and constitutively expressed *vir* genes in their dissection of the super binary vector pSB1. In their study, pSB1 was split into a pure T-DNA vector—with a backbone consisting of only replication machinery and antibiotic resistance—and pairs of pVIR accessory vectors that harbored different parts of the *vir* operons from the hypervirulent pTiBo524 binary vector and a constitutive VirG mutant. This ternary vector system was tested in different *A. tumefaciens* strains in combination with the expression of the *BABY BOOM* and *WUSCHEL* genes, leading to a transformation frequency of 83-103% and single copy frequencies of up to 34% in the PH2RT maize inbred line [114]. In a follow-up study, the system was applied in high efficiency transformation of sorghum variety Tx430 with a transformation frequency of 29.4% and single copy events (SC) at 54% [115].

**Table 2:** Recent binary/ternary vector approaches for enhancement of single copy, backbone-free events during AMT. Transformation frequency: number of selectable transformants; single-copy frequency: ratio of selectable transformants with single T-DNA integrations; backbone free: ratio of single copy transformants without backbone integration.

Approach	Agro strain/helper plasmid	Engineered plasmid	Organism	Transformation frequency (%)	Single-copy frequency (%)	Backbone free (%)	Source
Backbone-coded lethal genes adjacent to LB	ABI/pMP90RK	pMON83916	*Glycine max*	2.3	83.9	67.6	[117]
Low copy oriR derivatives	ABI/pMP90RK	oriRi5.6/pMON83935	*Glycine max*	1.0	33.8	86	[113]
	ABI/pMP90RK	oriRi4.2/pMON83937	*Brassica napus* L.	11.4	52.5	97	
	ABI/pMP90RK	oriRi4.2/pMON97352	*Zea mays* L.	9.5	60.6	95	
Strain & plasmid copy number optimization	AGL0/pTiBo542	PHP51901	*Zea mays* L.	75.8	31.6	55.1	[112]
	LBA4404/pAL4404	PHP51901	*Zea mays* L.	63.2	31.7	73.4	
T-DNA launched from chromosome	GV3101/pMP30	pTF101.1	*Arabidopsis thaliana*	2.11	78	97	[116]
	GV3101/pMP30	pTF101.1	*Zea mays* L.	0.9	64	92	
Ternary vector system	LBA4404 THY-/pAL4404	pPHP71539	*Zea mays* L.	31.1	29.9${}^{*}$	29.9${}^{*}$	[114]
	LBA4404 THY-/pAL4404	pSB1	*Zea mays* L.	13.7	36.6${}^{*}$	36.6${}^{*}$	
Ternary vector system	LBA4404 THY-/pAL4404	pPHP71539/pPHP78152	*Sorghum bicolor*	29.4	54${}^{*}$	100${}^{*}$	[115]
	LBA4404 THY-/pAL4404	pPHP71539/pPHP78233	*Sorghum bicolor*	24.8	65${}^{*}$	100${}^{*}$	
Coexpression of octopine and succinamopine *vir* genes	EHA105/pTiEHA105	pDAB112807/pDAB111437	*Zea mays*	13.4	97.5	90.1	[118]

${}^{*}$In the study, only single-copy events without backbone integration were monitored, thereby leading to 100% backbone-free events.

Two elegant solutions for higher SC and backbone-free transformants were explored: (1) launching the T-DNA from the bacterial chromosome (2.1% transformation frequency, 78% SC, and 97% backbone free) and (2) including a lethal gene cassette on the binary vector backbone next to the left border sequence (2.3% transformation frequency, 83.9% SC, and 67.6% backbone free) [116, 117]. These approaches did not outperform the transformation frequency of high plasmid copy number approaches, but depending on the aim of the study, low transformation frequency with high SC and backbone free frequency can outperform high transformation frequency, as high transformation frequency often does not correlate with SC events (Table 2). A study that mitigates this problem is the approach of Sardesai et al. [118]. In their work, octopine and succinamopine *vir* genes were coexpressed in a ternary vector system with the T-DNA flanked by three left border repeats, enhancing nicking efficiency and boosting the SC event rate to 97.5% with 90.1% backbone-free transformants and a transformation frequency of 13.4%. This work showcased the significant impact of the left border nicking on both SC without backbone integration.

A thoughtful binary or ternary vector design can significantly enhance desired T-DNA integration modification and reduce the time lost from failed experiments in slow turnover plant stable line experiments. Importantly, the precise control of *vir* gene expression could be essential to the understanding of the impact of different *vir* gene titers on host T-DNA titers, the transformation frequency, SC, and backbone free transformants.

### 4.2. *Agrobacterium* Synthetic Biology and Chassis Engineering

In comparison to the sophisticated synthetic biology toolboxes available in model bacteria, there has been little rigorous characterization of genetic parts in *A. tumefaciens* despite years of study and the development of many variants of binary vectors. At the time of publication, there have been few attempts to characterize the relative strengths of constitutive promoters in *A. tumefaciens* in a standardized manner; additionally, few examples exist of well-characterized inducible systems. An early work demonstrated that both *araC*- and *lacI*-based induction systems do work in *A. tumefaciens* [119], though the level of characterization was limited. A *tetR* system has also been used in *A. tumefaciens* to control the expression of *cre*-recombinase [120]. A later work by Khan et al. showed that *P_lac_* can generate ~20x induction over background as measured by the Miller assay and is titratable by IPTG in *A. tumefaciens* C58 [121]. A more comprehensive analysis of IPTG and cumic acid inducible systems in *A. tumefaciens* was done by examining the ability of engineered inducible promoters to complement a *virE2* deletion when infecting tobacco plants [122]. Measuring the activity of promoters via Miller assay as well as efficiency of agroinfiltration, the authors found that in AT *P_lac_* was inducible but *P_tac_* was constitutive. However, a *P_T5/lacOlacO_* promoter was shown to be 15x stronger than *P_lac_* by Miller assay and was 50x inducible above background. Interestingly, the authors showed that a *lacI*-regulated *T7* system was nonfunctional in AT, though a previous group had shown functionality [123]. A cumic acid regulated *P_T5_* promoter showed 50x the expression strength of *P_lac_* and showed 20x induction over background. However, the cumic acid regulated *P_T5_* also showed 10x higher basal expression relative to the *P_T5/lacOlacO_* promoter. A cumic acid-inducible *P_tac_* promoter showed comparable expression strength, but only half of the basal activity. While these works are an excellent starting point, there still remains great work to do in the development of reliable genetic control in *A. tumefaciens*.

In addition to the relative dearth of characterized genetic parts for *A. tumefaciens*, there has also been relatively little rational engineering of its genome to increase its ability to transform plants. Plants that produce ethylene had been known to be more resistant to *A. tumefaciens* infection, which was later shown to be caused by ethylene-mediated downregulation of the *vir* genes [124]. The same group then introduced 1-aminocyclopropane-1-carboxylate (ACC) deaminase—which cleaves the precursor of plant ethylene—into *A. tumefaciens*, increasing gene transfer into the ethylene producing melon *Cucumis melo* L. var. *cantalupensis* cv. Vedrantais [124]. Subsequently, Nonaka et al. suggested that *γ*-aminobutyric (GABA) may also inhibit T-DNA transfer, showing that introduction of the GABA transaminase *gabT* into *A. tumefaciens* moderately increased gene transfer into two tomato cultivars and the bioenergy crop *Erianthus arundinaceus* [125]. In both tomato and *Erianthus ravennae*, introduction of both ACC deaminase and *gabT* into *A. tumefaciens* resulted in increased T-DNA relative to when either of these genes was expressed individually [28]. Cysteine auxotrophic strains of *A. tumefaciens*—derived from *Tn5* insertion mutants—have not only shown the ability to prevent plant suppression of *vir* gene expression but also resulted in an 85-fold increase in transient GUS expression in *Nicotiana glutinosa* tissue cultures [126]. A patent for using thymidine auxotrophic *A. tumefaciens* has also been filed, for the purposes of both biocontainment and more efficient genetic transformation [127]. A work that further explores the metabolic and physiological functions of *A. tumefaciens* and how these are needed to thrive in the plant-host environment will continue to provide targets for rational engineering of the bacterium.

### 4.3. Disruption of Host Response and Induction of Host Regeneration

Two major hurdles to recovering successfully transformed plants are (1) the selection of positive transformants and (2) plant regeneration of transformed cells. The selection of transformed plant tissues is typically done by antibiotic or herbicide resistance but can also be done through the expression of a marker gene. In either case, selection depends on transgene expression, which can be strongly influenced by the induction of the siRNA pathway and epigenetic silencing of integrated transgenes [97, 128, 129]. Suppression of the siRNA response, either through coexpression of viral silencing suppressors [130] or through targeted down regulation of DCLs or RNA-dependent RNA polymerases, greatly increases the efficiency of transgene expressing plant tissue [97].

Despite the multitude of barriers to *A. tumefaciens* infection and transgene expression, plant cell regeneration is perhaps the most formidable obstacle limiting plant transformation [131]. In many cases, agronomically important genotypes are recalcitrant to plant tissue culture and callus regeneration. For this reason, specific plant genotypes with either increased plant regeneration properties or somatic embryo formation are used [131]. Often, these genotypes are agronomically suboptimal and integrated transgenes of interest must be crossed into elite cultivars followed by successive backcrosses to limit the genome drag of undesirable traits. Recently, breakthrough technology using transcription factors controlling cell proliferation and meristematic identity has been successfully used to increase the regeneration and transformation efficiency of recalcitrant plant species and genotypes [132, 133]. Adding to this work, Maher and colleagues have recently shown that combinatorial expression of WUSCHEL, MERISTEMLESS, and BABYBOOM was able to initiate efficient meristem induction *de novo*, resulting in the outgrowth of miniature plantlets [134]. This is a major advance, as it circumvents the time-consuming and labor intensive process of tissue culture and somatic embryo development.

## 5. Future Directions

**Figure 1:**
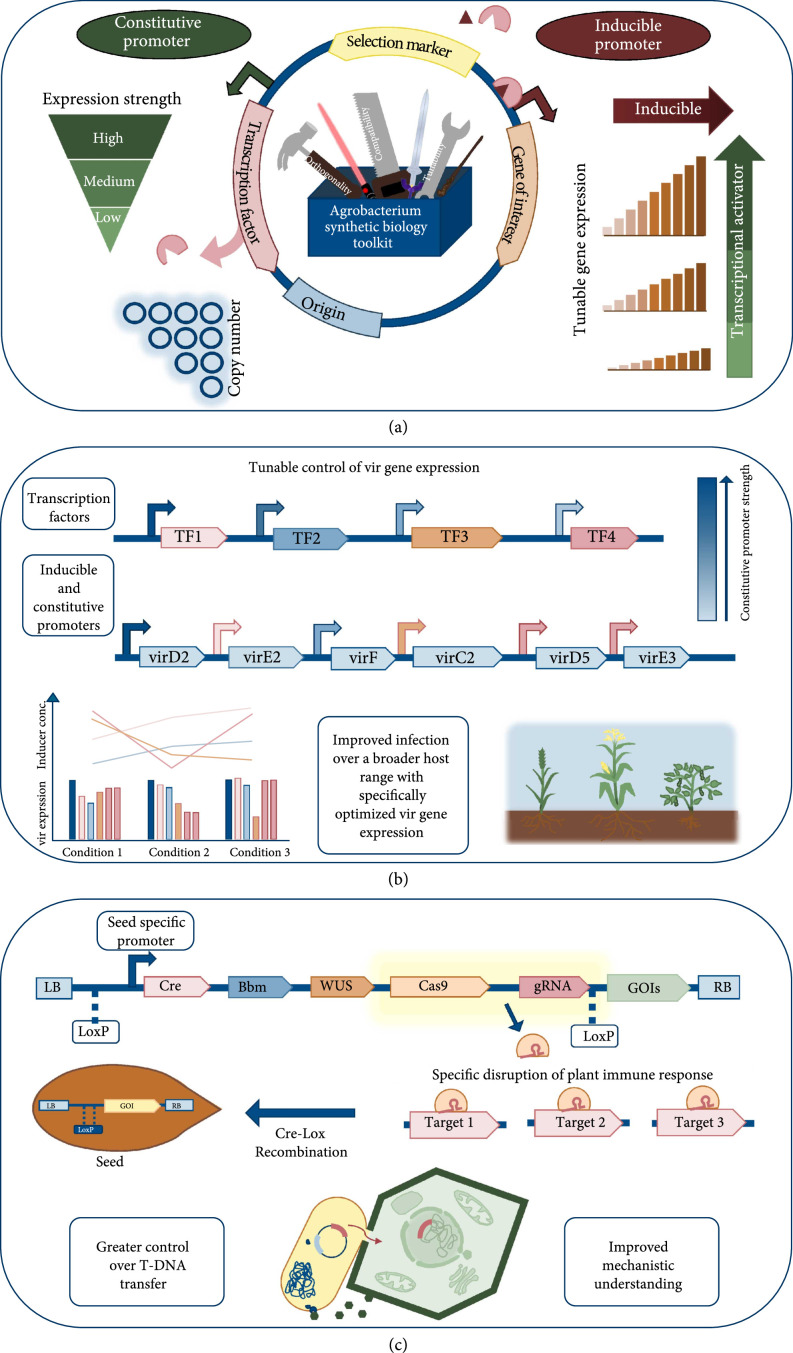
Future steps to improve AMT via synthetic biology: (a) development of a synthetic biology toolkit for *A. tumefaciens* will require the characterization of both constitutive and inducible promoters. Inducible promoter systems will need to have the dose response of inducers tested, in addition to precise titration of TF expression. Toolkits will also require distinct compatible origins of replication that should vary in copy number. (b) Once in place, *vir* genes can be precisely expressed to achieve desired titers of key virulence genes. Depending on the desired transformation outcome and host plant, the virulence complement can be engineered to achieve specific goals. (c) Complimentary synthetic biology tools encoded by transferred T-DNA will further modulate host gene expression and plant regeneration. Catalytically inactive or mRNA-targeted Cas9 variants can be used to silence multiple host genes without generating heritable DNA editing. In parallel, expression of Bbm and WUS increases plant regeneration and can be combined in tandem with other host regulatory proteins to manipulate pathogenesis. Expression of Cre recombinase under the control of a seed-specific or chemically inducible promoter excises T-DNA regions flanked by loxP sites to generate marker-free plants in the T1 generation.

Through many technical innovations, *A. tumefaciens* can now be used to engineer the genomes of many plants well beyond its natural host range. While these developments are substantial, there has been a lack of precision genetic control in nearly all currently published binary or ternary systems. A critical first step remains in developing a robust *A. tumefaciens* synthetic biology toolkit of well-characterized constitutive and inducible promoters, terminators, and origins of replication (Figure 1(a)). There are now many examples of excellent toolkits being constructed for nonmodel bacteria such as *Pseudomonas putida* [135], *Vibrio natriegens* [136], *Streptomyces venezuelae* [137], and Cyanobacteria [138]. Once a toolkit is in place, the virulence plasmid would be able to be entirely synthetically refactored (Figure 1(b)). This would allow researchers to titrate the expression of specific virulence factors to engineer desired transformation outcomes (i.e., high transient T-DNA expression or increased host range). Furthermore, such precise genetic control would allow researchers to elegantly interrogate basic questions of *A. tumefaciens* virulence [139].

Similar synthetic biology principles can also be applied to interrogate plant host responses that influence plant transformation and regeneration. Combinatorial knockdown and overexpression of host genes may help tailor plant transformation to particular plant species or genotype, while also providing tools to probe the underlying host biology of *A. tumefaciens* pathogenesis (Figure 1(c)). Moving forward, exerting simultaneous, precise control over both host and pathogen systems may usher in a new era of agricultural biotechnology.

## Data Availability

All data is freely available upon request.
